# Three-month outcomes from a patient-centered program to treat opioid use disorder in Iowa, USA

**DOI:** 10.1186/s13011-021-00342-5

**Published:** 2021-01-12

**Authors:** Alison C. Lynch, Andrea N. Weber, Suzy Hedden, Sayeh Sabbagh, Stephan Arndt, Laura Acion

**Affiliations:** 1 Department of Psychiatry, University of Iowa, Iowa City, IA USA; 2 Department of Family Medicine, University of Iowa, Iowa City, IA USA; 3 Department of Internal Medicine, University of Iowa, Iowa City, IA USA; 4 Instituto de Cálculo, Universidad de Buenos Aires – CONICET, Buenos Aires, Argentina

**Keywords:** Opioids, Addiction recovery, Treatment retention

## Abstract

**Background:**

Opioid use disorder (OUD), a chronic disease, is a major public health problem. Despite availability of effective treatment, too few people receive it and treatment retention is low. Understanding barriers and facilitators of treatment access and retention is needed to improve outcomes for people with OUD.

**Objectives:**

To assess 3-month outcomes pilot data from a patient-centered OUD treatment program in Iowa, USA, that utilized flexible treatment requirements and prioritized engagement over compliance.

**Methods:**

Forty patients (62.5% female: mean age was 35.7 years, SD 9.5) receiving medication, either buprenorphine or naltrexone, to treat OUD were enrolled in an observational study. Patients could select or decline case management, counseling, and peer recovery groups. Substance use, risk and protective factors, and recovery capital were measured at intake and 3 months.

**Results:**

Most participants reported increased recovery capital. The median Assessment of Recovery Capital (ARC) score went from 37 at enrollment to 43 (*p* < 0.01). Illegal drug use decreased, with the median days using illegal drugs in the past month dropping from 10 to 0 (*p* < 0.001). Cravings improved: 29.2% reported no cravings at intake and 58.3% reported no cravings at 3 months (p < 0.001). Retention rate was 92.5% at 3 months. Retention rate for participants who were not on probation/parole was higher (96.9%) than for those on probation/parole (62.5%, *p* = 0.021).

**Conclusion:**

This study shows preliminary evidence that a care model based on easy and flexible access and strategies to improve treatment retention improves recovery capital, reduces illegal drug use and cravings, and retains people in treatment.

## Background

The United States has experienced a dramatic increase in opioid use. Deaths due to opioid-related overdose have risen across the country over the past 20 years, with over 47,000 overdose deaths involving an opioid in 2017 [1]. After falling slightly in 2018, provisional data suggest that opioid-related overdose deaths rose to a new high in 2019, with over 50,000 Americans dying from an opioid overdose [2]. Overdose deaths appear to continue rising since the Covid-19 pandemic began. Data from the National EMS Information System (NEMSIS) show that overdose-related cardiac arrests have risen sharply [3]. Provisional data from the US Centers for Disease Control and Prevention indicates that overdose deaths are on pace to reach a new all-time high in 2020 [4].

In 2017, the United States Department of Health and Human Services declared the Opioid Crisis a public health emergency and recommended a 5-point strategy to combat it: 1) Improve access to prevention, treatment, and recovery support services; 2) Target the availability and distribution of overdose-reversing drugs; 3) Strengthen public health data reporting and collection; 4) Support cutting-edge research on addiction and pain; and 5) Advance the practice of pain management [5]. A critical component of the nation’s response to this devastating epidemic is improving access to treatment and recovery services. Access is a key point because effective treatment exists, but it is underutilized. Data from the National Survey on Drug Use and Health [6] show that fewer than 20% of people who identify as having an OUD get treatment.

Access to treatment is important because it enables people with OUD to get connected to a care provider, to receive medications and treatment recommendations, to develop goals and a plan for ongoing management, and hopefully to remain in treatment. Staying in treatment is important: a recent study showed that medication for addiction treatment (MAT) reduced mortality rates for people with OUD by 80%, but this benefit occurred only as long as the person remained in treatment [7].

Treatment engagement is critical in order to reduce risk of returning to drug use and potential mortality. Identifying and adjusting barriers that prevent people from accessing or staying in treatment could improve treatment rates. Rigid rules for treatment, such as requiring psychotherapy or terminating treatment if the individual has any drug use, are barriers for treatment access and retention [8]. Despite the importance of treatment retention, a systematic review of 55 articles from randomized controlled trials looking at treatment retention with MAT found a wide variability in treatment retention rates, with 6-month treatment retention rates ranging from 3 to 88% [9].

Successful treatment should include approaches to enhance treatment retention and low-barrier pathways for re-entering treatment when recovery has been interrupted. As OUD is a chronic health condition, symptom recurrence is common and too often leads to substance reuse and treatment termination. A person with diabetes who eats a candy bar or has an elevated hemoglobin A1c would likely receive dietary counseling and a medication adjustment, but a person in treatment for a SUD is more likely to be discharged from treatment if they use a substance or have a positive urine drug test [10]. Understanding the components of treatment and recovery services that increase treatment retention and reduce barriers to treatment re-entry are needed to improve outcomes for people with OUD.

In this study we sought to assess 3-month outcomes from a patient-centered practice that included MAT with buprenorphine or naltrexone plus the option to participate in psychosocial treatments. The psychosocial treatments included case management, psychotherapy, peer recovery groups such as Narcotics Anonymous or Smart Recovery, or peer support through a local harm reduction program. We hypothesized that patients were more likely to remain in treatment if participation in one or more psychosocial treatments was optional rather than required. We also hypothesized that allowing patients to continue in treatment even if they used a substance or had a relapse would increase treatment retention.

## Methods

### Participants

Study participants (*n* = 40) were recruited from the University of Iowa (UI) MAT Clinic. The UI MAT Clinic is located in an academic medical center in Iowa City (Iowa) and provides outpatient care to adults who have OUD and who are receiving MAT with buprenorphine or naltrexone.

This study’s inclusion criteria were people who have OUD and are receiving MAT with buprenorphine or naltrexone; age 18 years and older; and able to speak English well enough to participate in case management and complete study surveys. Not intending to continue care in the clinic for at least the next 6 months was the exclusion criterion. Case managers approached patients who were eligible and invited them to participate in the study.

### Setting

Study participants were self-referred to the UI MAT clinic or referred by a provider or after presenting to the hospital’s emergency department (ED) in opioid withdrawal and starting buprenorphine. In addition, UI MAT clinic staff partnered with community representatives from law enforcement, community corrections, social service agencies, healthcare providers, and a harm reduction organization to promote referrals to the clinic. The providers in this clinic included physicians, nurses, social workers, addiction counselors, and case managers. In addition, resident physicians from psychiatry and family medicine rotated in this clinic, spending between 1 and 12 months on an addiction medicine rotation.

**Fig. 1:**
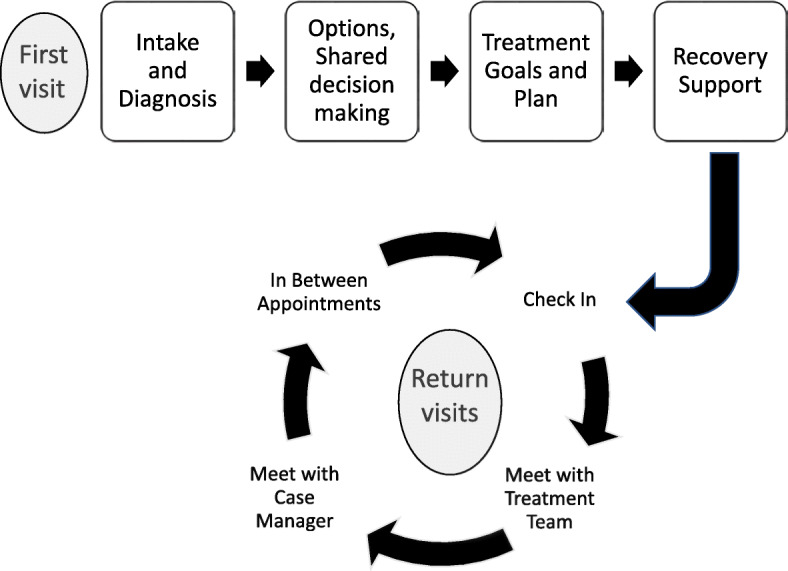
During the first visit to the clinic, patients undergo an intake and diagnosis, discuss and select treatment options, set goals, and work with a case manager to develop a recovery plan. Patients meet with the case managers at each visit and communicate with the case manager in between appointments

As seen in Fig. 1, patients initiating care at the UI MAT Clinic underwent a diagnostic evaluation, which included a complete medical, psychiatric, and substance use history, completion of the Brief Addiction Monitor [11], an examination, diagnostic determination, discussion of options including risks and benefits, shared decision making and development of a treatment plan. Patients with OUD were offered MAT with buprenorphine or naltrexone; a prescription for naloxone and instructions on how to use it to treat opioid overdose; and linkage with a clinic case manager. Patients also received information about local resources including counseling, mutual support groups, and a harm reduction organization. Patients met with a case manager at the end of each clinic visit to discuss treatment goals and recovery supports, and to schedule follow up appointments. In between appointments, case managers were available by phone or text so patients had easy access if they had questions or experienced unexpected developments (e.g. change in health insurance, housing, or transportation) that could interfere with treatment adherence.

Clinic staff saw patients weekly at the beginning of treatment. The duration between follow-up visits was gradually increased as their recovery stabilized. At each visit, the treatment team assessed risk for return to drug use, HIV, hepatitis C, pregnancy, medical and psychiatric co-morbidities, and overdose. When indicated, testing, referral, prevention, and treatment were offered.

When a patient missed an appointment, the case manager called or texted them to find out what happened, to assess for medication refill needs, and to assist with rescheduling. Maintaining medication adherence was prioritized. If a patient missed multiple appointments, the case manager worked with them to identify and overcome barriers to attending appointments. The clinic had a written policy stating patients could be dismissed from the clinic if they did not attend appointments or if they engaged in problematic behavior such as threatening staff or selling their MAT medication, however this policy rarely needed to be utilized.

Patients could participate in additional services at the MAT clinic, including the dual diagnosis partial hospital program, the intensive outpatient program, counseling, or other relapse prevention services. Mutual support groups such as Narcotics Anonymous and Alcoholics Anonymous were available in the hospital and at nearby locations. The Clinic provided information about local meeting times and places and encouraged using this support if patients believed it was helpful to their recovery. Patients received information about a local harm reduction organization that offered peer support services as well as harm reduction services (e.g. naloxone distribution, fentanyl test kits for drugs, hepatitis C testing). The harm reduction organization also referred patients to the clinic.

Several evidence-based practices were incorporated into the care offered in the MAT clinic, to supplement the medications for addiction treatment. All providers had training in motivational enhancement therapy, and this approach was utilized during evaluation, treatment planning, and monitoring at follow up visits. When patients were not meeting treatment goals, additional treatment options were discussed, such as adding counseling or increasing the level of care.

It was expected that patients would continue receiving MAT and participating in the MAT clinic for as long as they found it beneficial to their recovery. Allowing patients to choose how long they continued to receive MAT was part of the patient-centered approach of the program. Some patients expressed an interest in tapering or discontinuing their medication as one of their goals; others intended to remain in treatment indefinitely. Either way, case managers and physicians supported each patient’s goals regarding treatment duration and assisted them in planning their recovery over the short and long term.

### Procedures and evaluations

This study was approved by the University of Iowa Institutional Review Board for Human Subjects Research. All study data were collected and managed using Research Electronic Data Capture (REDCap) tools hosted at the University of Iowa [12, 13].

**Fig. 2:**
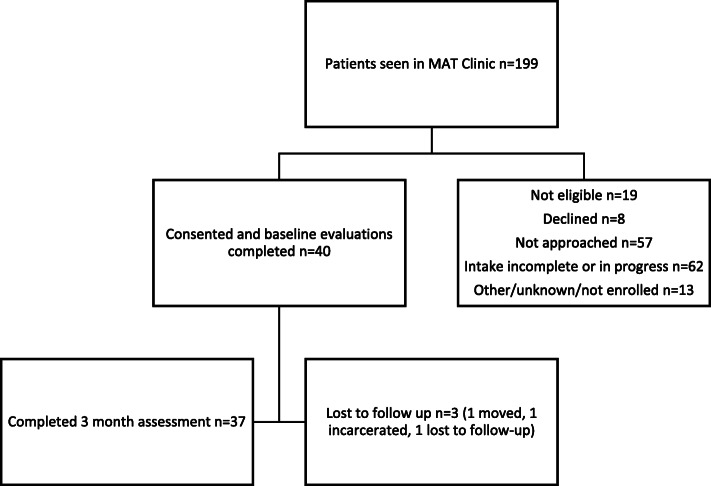
During the study period, 199 unique individuals were seen in the clinic. Of these, 40 were enrolled in the study, and 37 completed the 3-month assessment

To recruit participants for this study, the case manager introduced the opportunity to participate to patients during a clinic visit. Patients were given a copy of the study consent form to review while they considered participating. If they decided to participate, the case manager reviewed the consent document and obtained informed consent. Patients who initially declined to participate in the study could opt to enroll at a subsequent appointment. After informed consent was obtained, the case manager enrolled the participant into the study. During the study period, 141 unique patients were seen in the clinic and 40 patients consented to participate in the study, as shown in Fig. 2.

Following enrollment, participants completed a thorough contact information form, the Government Performance and Results Modernization Act of 2010 [14] (GPRA) tool, a previous drug use survey, the Brief Addiction Monitor (BAM) [11] to assess recent drug use, risk factors for relapse, and protective factors for recovery; and the Assessment of Recovery Capital [15] (ARC).

Next, the case manager reviewed the ARC scores with the study participant and selected 2–3 domains with the lowest scores to guide selection of resources and services to be provided during treatment.

Throughout participation in the study, participants continued to receive treatment for OUD in the MAT clinic. Some participants opted to work with an addiction counsellor who provided Cognitive Behavior Therapy (CBT), Motivational Interviewing (MI), and/or 12-Step Facilitation. Study participants who, at any point, were not meeting treatment goals and had barriers to accessing counseling were invited to utilize a web-based counseling program, Computer Based Training for Cognitive Behavior Therapy (CBT4CBT) [16]. This self-guided, interactive web-based program has been shown to teach cognitive behavioral skills for reducing and managing substance use. In order to receive CBT4CBT, participants had to have access to a computer or smartphone with internet capabilities; the cost of the program was covered by a grant.

Three months following enrollment into the study, the case manager met with each participant to complete a care coordination form, list services that had been provided over the past 3 months and identify which services would be provided in the coming 3 months. The care manager re-administered the GPRA, BAM, and ARC assessments. The participant also completed a Service and Satisfaction Scale that was developed for this project, to obtain feedback about the care and services received.

### Statistical methods

Data are described using medians and proportions. Categorical data were analyzed using the Fisher exact test and McNemar’s test for before and after comparisons. Baseline and 3-month continuous outcomes were compared using the exact Wilcoxon signed-rank test.

All analyses were performed using R 3.6.1 [17] and R packages janitor [18], tidyverse [19], and coin [20]. All the *p*-values reported are 2-tailed. Significance level was set at *p* < 0.05.

## Results

### Participants characteristics

A total of 40 participants out of whom 25 (62.5%) self-identified as females participated in the study. The participants had a mean age of 35.7 years (sd = 9.5 years). The youngest participant was 22 and the oldest was 64 years old. Racial, ethnic, and education characteristics are detailed in Table 1. About a quarter of the sample (*n* = 11, 27.5%) resided in rural Iowa counties, 32.5% (*n* = 13) were employed full-time, 12.5% (*n* = 5) were employed part-time, and 52.5% (*n* = 21) were unemployed. At baseline, 52.5% (*n =* 21) participants rated their overall health as good or very good and 19 participants as fair or poor. None of the participants indicated their overall health was excellent. Of the 40 study participants, 19 received psychiatric treatment from their MAT provider.

**Table 1:** Participant characteristics

Race	n	%
American Indian or Alaska Native	1	2.5
Asian	1	2.5
Black or African American	5	12.5
Hispanic or Latino	1	2.5
White	32	80.0
**Education**		
Less than High School	5	12.5
High School	11	27.5
Some College	18	45.0
Bachelor’s	3	7.5
Vocational or Technical Diploma	3	7.5

### Treatment outcomes

Thirty-seven (92.5%) participants were retained in treatment for 3 months. Retention rate was significantly higher for participants who were not on probation or parole (31 out of 32 participants retained, 96.9% 3-month retention rate), when compared to participants on probation or parole (5 out of 8 participants retained, 62.5% 3-month retention rate, Fisher Exact test *p* = 0.021).

### Addiction-related outcomes

**Fig. 3:**
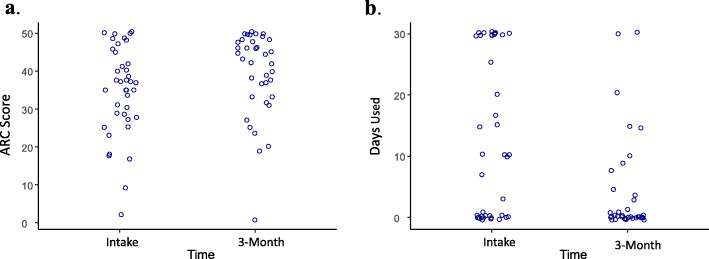
**a**: ARC scores for baseline and 3-month visits. **b:** Past 30-day illegal drug use at treatment intake and 3-month visits

For the participants that completed 3 months of treatment (*n* = 37), ARC scores improved at the 3-month visit. The median ARC at intake was 37, while the median at 3-months was 43 (Exact Wilcoxon Signed-Rank Test z = − 2.6, *p* < 0.01, Fig. 3a).

When past 30-days use of illegal drugs was evaluated, there was a significant reduction in the number of days during which participants used these substances. The median number of days participants used illegal drugs at intake was 10 days versus 0 day at 3-months (Exact Wilcoxon Signed-Rank Test z = 3.2, *p* < 0.001, Fig. 3b).

Of the 37 people with both intake and 3-month thirty-day abstinence for illegal drugs, 15 (40.5%) were abstinent at intake. At 3-month follow-up, 23 (62.2%) were abstinent (Exact McNemar’s Test chi-squared = 5.3, *p* = 0.039).

**Fig. 4:**
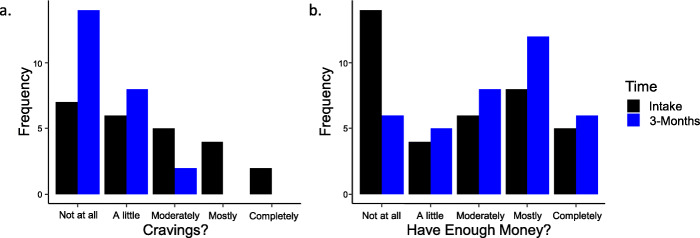
**a**: Past 30-day cravings at treatment intake (black bars) and 3-month visits (blue bars). Almost a third of participants answered that they had no cravings at intake. That amount doubled after 3 months of treatment. **b**: Participants at 3 months considered they had enough money to meet their needs more often than at treatment start

Due to a delay in implementing the BAM among assessments, from the 37 patients who completed the 3 months of treatment, there were only 24 participants with the BAM data at intake and 3 months. For the BAM’s question “In the past 30 days, how much were you bothered by cravings or urges to drink alcohol or use drugs?”, there was a significant reduction in past 30-day craving between the intake and 3-month visits. Of the 24 people with complete data, at intake, 29.2% answered “Not at All” when asked about cravings at intake while, at 3-months, 58.3% had no cravings (Exact Wilcoxon Signed-Rank z = 3.54, *p* < 0.001, Fig. 4a).

## Discussion

This study shows that people with OUD can achieve a high treatment retention rate, cut down their use of illicit drugs, and build their recovery capital. More than half (62.2%) of the study participants were abstinent from substance use during this study, but some (37.8%) participants used opioids or other substances during the study. Continued use was not a criterion for terminating treatment. As expected, this patient-centered program had a high rate of treatment retention, with, over 90% of participants in this study retained in treatment for OUD at 3 months. During their treatment, participants reported fewer cravings to use opioids and reduced use of other illicit drugs. In addition, patients reported significant improvement in their ability to access the personal, family and community resources needed to find and maintain recovery, also called recovery capital.

Treatment retention with MAT is important because it has been linked to substantial reductions in both all-cause and overdose-related mortality in people with OUD [21]. Furthermore, treatment retention is essential, since discontinued care increases the risk of overdose. Understanding what optimizes treatment retention is critical to implementing programs that are successful in keeping people in treatment. In a systematic review looking at OUD treatment retention, MAT with methadone, buprenorphine, or naltrexone was highly associated with increased treatment retention [9]. In this review, retention rates across different treatment settings were extremely variable. Between 19 and 94% of people who initiated MAT for OUD remained in treatment at 3 months, with an average of 62% retained at 3 months across studies. At 92.5%, our study’s 3-month retention rate is comparable to the highest performing studies in the systematic review.

The role of psychosocial or behavioral treatment in promoting treatment retention in office-based settings is unclear, as there are few studies examining this issue and some studies have reported conflicted findings. A systematic review of 8 randomized clinical trials evaluating MAT with buprenorphine, with or without various behavioral interventions, found 4 studies showed benefit and 4 studies did not [22]. Three of the four studies that found benefit used a contingency management-based intervention [23–26]. While there may be some value added by offering psychosocial and behavioural treatments with MAT for OUD, and some patients may wish to incorporate these treatments into their overall care plan, current data do not support requiring its use.

In patient-centered care, healthcare decisions and outcome measures are selected to meet the patient’s health needs and desired health outcomes [27]. Incorporating more patient-centered approaches in addiction treatment could increase treatment engagement and retention [28]. Two recent reports examine patient-centered changes in OUD treatment protocols that were implemented in response to the Covid-19 pandemic, including more treatment flexibility with take-home doses and operating with less certainty due to less access to urine drug testing [29, 30]. More research is needed to better understand how patient-centered care can impact outcomes for OUD. Our study used a patient-centered approach, incorporating shared decision making and personalized treatment plans. Participants were offered psychosocial or behavioural treatments such as cognitive behaviour therapy-based group psychotherapy and peer support groups, and motivational enhancement therapy was utilized during medication management to assist with overcoming barriers to recovery, but these interventions were not a required component of treatment.

Mental illness (MI) frequently co-occurs with SUDs and is a risk factor for treatment non-completion and early departure from treatment [31]. Integrating psychiatric treatment with SUD treatment leads to improved outcomes in general, and may lead to higher rates of treatment completion, compared to treating SUD and MI separately [32]. For patients with OUD who are receiving MAT, MI does not appear to have a measurable impact on OUD treatment retention [33], but integrated treatment for MI and OUD improves MI treatment initiation and mental health outcomes [34]. In our study, most of the MAT providers were also psychiatrists, and study participants could receive integrated treatment for co-occurring MI if needed. More research is needed to determine if patients benefit from integrated MI and SUD treatment.

Among people with a SUD, ongoing substance use is a risk factor for adverse health outcomes [35], so it stands to reason that reducing or stopping substance use would improve health. People who cut back or stop using substances have less adverse consequences of drug use such as mental health symptoms or impairment [36], have improved social and family functioning [37], and are less likely to engage in criminal behaviour [38]. In a study of outcomes for people with OUD, reductions in regular heroin use were strongly associated with reductions in crime [38]. Other studies have shown that reductions in cocaine use are associated with reductions in crime [39, 40]. Reducing substance use has been shown to improve adolescents’ school attendance [41]. Because of its positive effects on health, reducing or abstaining from substance use is a treatment target for people with OUD. In our study, 40.5% of participants reported no substance use for the past 30 days at intake, and that increased to 62.2% at 3 months. Importantly, while 30-day abstinence from substance use increased over the 3-month period, persistent substance use did not prompt treatment termination. Applying a chronic disease model approach, when symptoms persist, the appropriate response is to continue or adjust treatment, not discharge from care. Furthermore, this approach also supports treatment retention. We followed this approach, which could be part of why the retention rate is in the high end for OUD treatment.

Cravings preoccupy the mind and distract from other thoughts and activities that can strengthen recovery. Cravings are hypothesized to play a central role in relapse to opioid use [42]. Reduced cravings is a primary treatment target when treating OUD, as cravings predict relapse of opioid use [43]. Buprenorphine, the medication prescribed to most patients at the UI MAT clinic, has been shown to significantly reduce cravings for opioids [43]. We titrated buprenorphine doses to control cravings. At intake, nearly a third of participants in our study reported no cravings to use opioids. By 3 months, the number of people reporting no cravings doubled.

It is unclear to what extent reducing cravings leads to increased retention versus retention in treatment drives reductions in cravings, although both effects are likely to play a role. Our study demonstrated a reduction in cravings and a high rate of treatment retention, but it was not designed to further characterize the relationship between these two outcomes.

Another component of treatment retention is the building of recovery capital. Recovery capital includes skills and attitudes related to confidence, self-efficacy, and support system. Recovery capital predicts sustained recovery, enhances life satisfaction, and enhances ability to cope with stress [44]. Early successes in recovery help to increase a sense of confidence in one’s ability to build their recovery. In our study, recovery capital, as measured by the ARC score, increased for most participants between intake and 3 months. Despite a decrease in the ARC score for 10 participants and a small sample size in our study, there was still a statistically significant increase in the mean ARC score overall. Over a 3-month period, our study found improvements in recovery capital, reduced cravings, and reduced substance use, all of which likely contribute to a high rate of treatment retention.

Treatment retention is irrelevant if there is no treatment access. Opioid-related deaths have risen dramatically in rural communities over the past decade and in 2015, the rural overdose death rate surpassed the urban overdose death rate. Yet access to MAT remains elusive for many rural communities, and there is a lack of studies on participants, treatment outcomes, and barriers to medication treatment for opioid use disorder in rural communities [45]. One of the strengths of our study was that a quarter of the participants live in rural communities. Further study could provide more information about factors that increase access and treatment retention for people with OUD who live in rural communities.

Additional ways to reach and engage rural and other populations who experience barriers to MAT include expanding treatment options and protocols, and policy changes. In Canada, people seeking MAT for OUD have more medications to choose from, including diacetylmorphine and hydromorphone. Access to these additional treatment options, both of which have both been shown to be effective and promote treatment retention in randomized controlled trials, could help more people in the US get into and stay in recovery [46–48]. Both of these medications can be administered intravenously, providing another approach to MAT that can be effective when oral agents are not [49]. Treatment protocols that are flexible and adjust to individual treatment goals without requiring participation in all aspects of treatment, such as talk therapy or 12-step programs, would remove a barrier to treatment for some. Expansion of MAT prescribing privileges to nurses, as has been done in British Columbia in Canada, and pharmacists would increase access by significantly increasing the number of available prescribers [50]. Innovative treatment delivery systems such as mobile care can provide a connection to OUD treatment for populations that have been historically harder to reach. Maintenance and expansion of the Affordable Care Act, enforcement of the federal Parity Act, and payment reform would also increase access to SUD treatment across the US [51]. And finally, applying a public health approach to address income disparities and other socioeconomic disadvantages, hopelessness and despair, stigma, systemic racism, income insecurity, access to employment and stable housing would address the opioid epidemic on a scale that medicine cannot [52].

### Limitations

Our results should be interpreted considering this study’s limitations. All study participants received treatment for opioid use disorder, but 40 % of participants in this study reported no illicit drug use in the 30 days prior to enrollment. Some patients transferred care from another provider and were already in recovery at the time of enrollment, and some patients chose to get started in treatment and then consented to study participation later.

This study was observational. There was no comparison group and we did not control for additional variables that could be influencing patient outcomes. Patients were offered a variety of services and were able to choose which services they received. The mix of services was not controlled for in this study so some services may be confounders for some patients. Some of the data gathered in this study was based on self-report and could be influenced by recall bias, incomplete or inaccurate information. Self-report tools are a component of the chronic care model [53] and measurement-based care [54], and they are a standard part of our clinical care, so we included these results in our study along with other more objective measures such as treatment retention.

We measured treatment retention and other outcomes for 3 months, but as treatment retention is associated with reduced mortality [7], treatment retention should be measured in years, not months. Longer duration of treatment, however, is built on a foundation of early treatment retention.

Our study showed high rates of treatment retention at 3 months. Treatment retention in the initial months of MAT is critical to achieving higher rates of treatment retention later. Several studies have demonstrated that treatment discontinuation is highest during the first month of treatment [55, 56]. Focusing on early treatment retention may help people with OUD to overcome causes of early treatment discontinuation and lead to higher rates of long-term treatment retention.

As a single-site study, reproducibility was not proven and effects sizes could be larger than those expected in a multi-site trial, but many of the components of the treatment program are widely available [57, 58] and can be replicated in other sites. Our sample does not reflect the US population, primarily because Iowa’s population is less diverse than the country’s overall. Our study population was majority female. While the study population has less racial and ethnic diversity than the general population, people living in rural areas are well-represented in this study. Access to SUD treatment and MAT is a challenge in many rural communities across the country so understanding the experience of care for people living in rural communities is relevant.

This study will continue to follow patients up to 3 years. The rather small sample size of 40 reflects recruitment during the first year for this study, follow-up publications will incorporate data from the whole sample. Since our early treatment retention rate was higher than usually reported, we consider these preliminary results can contribute significantly to the OUD treatment field. Our study supports previous findings showing that evidence-based treatment (in particular MAT), offering but not requiring less proven treatment approaches (e.g., psychosocial treatment), and continuing treatment even when all treatment goals (e.g., 30-day substance use abstinence) are not met continuously can achieve high rates of success with treatment retention and other associated outcomes.

### Next steps

Our study demonstrates that high rates of treatment retention during the first 3 months of recovery are achievable. Further study is needed to determine which variables improve early treatment retention, whether these increased rates of treatment retention can be sustained during the later stages of recovery, and whether these results can be replicated in other sites and patients with different characteristics. Further disentangling the mechanisms behind optimizing recovery and treatment retention is warranted.

## Conclusions

This study demonstrates that people receiving MAT for OUD can have high rates of treatment retention, reduce their substance use, and build recovery capital. Factors that likely contribute to treatment success include prioritizing treatment retention, using patient-centered treatment planning, and integrated treatment for co-occurring disorders. Increased utilization of MAT and interventions that enhance treatment retention and recovery are critical to ending the opioid crisis.

## Data Availability

The datasets analysed during the current study are available from the corresponding author on reasonable request.

## Abbreviations

OUD: Opioid use disorder
SUD: Substance use disorder
ARC: Assessment of Recovery Capital
SAMHSA: Substance Abuse and Mental Health Services Agency
DEA: Drug Enforcement Agency
MAT: Medications for Addiction Treatment
UI: University of Iowa
ED: Emergency Department
REDCap: Research Electronic Data Capture
GPRA: Government Performance and Results Act
BAM: Brief Addiction Monitor
CBT4CBT: Computer Based Training for Cognitive Behavior Therapy
MI: Mental Illness
